# American Academy of Dental Science

**Published:** 1876-11

**Authors:** 


### ARTICLE V.

### *Annual Meeting of the American Academy of Dental Science, Boston.*

We are indebted to Dr. E. N. Harris, of Boston, for the following account of the proceedings of this excellent society:

The Ninth Annual Meeting of the American Academy of Dental Science was held in the Union Hall, 18 Boylston Street, yesterday, Dr. D. M Parker, presiding. The records of the last annual and monthly meetings were read by the recording secretary, Dr. W Lewis Tucker., and approved. The Treasurer, Dr. L. D. Shepard, reported all bills paid and a surplus left in the treasury. The Centennial Committee reported that they had prepared a history of dentistry in America during the past one hundred years, which had been published, making a handsome volume of 300 pages. It had been received with great interest by the profession throughout the country. Its preparation had involved great labor, and expense to the Academy of about $1500. This expense is a free gift to the profession.

The censors reported the names of Dr. M. S. Dean, of Chicago, Dr. G. H. Cushing, of Chicago, Dr. W. A. Bronson, of New York, Dr. John W. Curtis, of Brunswick, Me., Dr. H. E. Smith, of Boston, and all were unanimously elected members of the Academy. The following officers were elected for the ensuing year:

President, Dr. D. M. Parker; Vice-President, Dr. E. G. Tucker; Corresponding Secretary, Dr. George T. Moffott; Recording Secretary, Dr. W. Lewis Tucker; Treasurer, Dr. L. D. Shepard, Librarian, Dr. John Clough; Board of Censors, Drs. J. L. Williams, W. W. Codman and E. N. Harris. A vote of thanks was given by the Academy to E. N. Harris, for the faithful and efficient manner in which he has discharged the duties of corresponding secretary during the past nine years. A committee of five was appointed to engage an orator and essayists for the next annual meeting, and the meeting adjourned until 2 o'clock P. M.

It was announced at the opening of the afternoon session that Dr. Robert Arthur, of Baltimore, who was to deliver the annual address, was unable to be present, but had sent the manuscript of the address, and this was read by Dr. Moffatt. The address was a plea for a more scientific study and practice of dentistry, in distinction of considering it merely as a manual art. The present age demands a broad view of the profession, and that its professors should rise above its mechanical routine, in order to meet the present wants and make it more of a science instead of considering it only as an art, as was the too universal practice. The need of more thoroughly educated men in the profession was urged, and the paper went on to define in what the scientific side of dentistry consisted. Most of its facts had been based upon experiment and had not been classed under theories. The question of dental caries was one in point. It was acknowledged that the subject of caries was becoming a more and more serious one, and their proper treatment was one of the highest tests of qualification of a dentist. The disease known as caries really gave dentistry its existence. But few and unimportant original investigations had been made in this direction in this country, most of such scientific knowledge coming from foreign investigators. Mechanical proficiency had become a test of professional status in this respect. Was this mechanical treatment of the disease to continue indefinitely? If so, the writer, could not make any claim for science in dentistry. The first duty of all conscientious dentists was to inculcate a more general knowledge among the young as to the care of their teeth. The doctor asserted that were such proper care observed, in the majority of cases the teeth could be kept free from caries, however much they might be predisposed to them. It might be that some agent would be discovered which, applied to the teeth, would prevent their decay, or some medicinal agent which would act to the same result through the system, or some way might be devised to cause a denser growth of the substance of the teeth. Such inquiries were of a much more scientific and important character than mere devotion to the matter of plugging caries of the teeth. It was well known that the greatest results in such scientific researches were often realized through the efforts of a few intelligent, studious devotees of a science, and such the writer believed was the province of the Academy. The essay closed with an appeal to the members to give these higher aspects of the science the attention they demand, without which the progress of intelligence would be retarded.

A vote of thanks was given Dr. Arthur for his able paper, and a copy was requested for publication. The reading of essays by members of the society was then in order. Dr. E. A. Bogue, of New York, was expected to read the first essay. but he was absent, and a letter from him was read, regretting that illness and family matters prevented his presence. Dr. Thomas Fillebrown, of Portland, then read an essay on "Specialties." The paper opened with a reference to the acknowledged importance of the division of labor in all callings. Dentistry was truly a specialty in medicine, and there were two theories as to what was the proper education for it. One was to consider it a profession by itself, and educate the aspirant solely for its prac tice; the other, to include it in the general study of medicine. The object of the paper was to advocate the latter course. As the dentist was called to treat patients under every variety of conditions to health, he was not worthy of the name of dentist if he was not able to understand these conditions and vary his treatment according to them. The writer closed by offering the following resolutions:

Whereas, Dentistry is a specialty of the science of medicine.

*Resolved,* That a thorough medical education is essential to the most successful practice of it.

*Resolved,* That we deem it expedient and for the best interests of the practice of dentistry, that existing medical schools enlarge their courses of instruction so as to include efficient instruction in the specialty of dentistry in order that it may be placed on an equality with other specialties of medicine.

The resolutions were temporarily laid on the table and the reading of the essays was proceeded with. Dr. C. A. Brackett, of Newport, read the next paper, his subject "Good Judgment an Essential Qualification for the Dentist." The importance of this qualification was illustrated by an enumeration of various contingencies liable to arise in the practice of every dentist where its absence would cause failure, oftentimes most disastrous. These illustrations were adduced to show that the subject might be pursued at an indefinite length. The paper was very suggestive and was heartily applauded.

Dr. E. W. Foster, of Boston, then read an essay "On the Oral Cavity and the Functions of the Teeth," this paper being substituted for one expected to be read by Dr. H. M. Bowker, of Montreal, who was absent. The paper, which was very interesting, considered the changes in the oral cavity, its range of function, the fifth nerve, the function of the teeth in song and speech, the tactile sense of the teeth, dental sensibility, and the power of the teeth to convey sound. At the close of this essay, discussions of the various papers was in order. Dr. Abraham Robertson, of Georgetown, strongly favored the adopting of the resolutions offered by Dr. Fillebrown, and supported the opinions expressed in Dr. Arthur's address. Dr. John Clough, of Woburn, Dr. H. F. Bishop, of Worcester, and other members took part in the discussion, which finally turned on the adoption of Dr. Fillebrown's resolutions. On being put to vote they were adopted unanimously. After voting thanks to the essayists, the society adjourned to meet at the Parker House, at 5 o'clock and partake of the annual dinner.

---

